# Recovery of transplantable organs after cardiac or circulatory death: Transforming the paradigm for the ethics of organ donation

**DOI:** 10.1186/1747-5341-2-8

**Published:** 2007-05-22

**Authors:** Joseph L Verheijde, Mohamed Y Rady, Joan McGregor

**Affiliations:** 1 Department of Physical Medicine and Rehabilitation, Mayo Clinic Hospital, 5777 East Mayo Boulevard, Phoenix, Arizona, 85054, USA; 2 Department of Critical Care Medicine, Mayo Clinic Hospital, 5777 East Mayo Boulevard, Phoenix, Arizona, 85054, USA; 3 Department of Biomedical Ethics and Medical Humanities Program, College of Medicine-Phoenix, University of Arizona and Department of Philosophy, Arizona State University, 300 East University Drive, Tempe, Arizona, 85287, USA

## Abstract

Organ donation after cardiac or circulatory death (DCD) has been introduced to increase the supply of transplantable organs. In this paper, we argue that the recovery of viable organs useful for transplantation in DCD is not compatible with the dead donor rule and we explain the consequential ethical and legal ramifications. We also outline serious deficiencies in the current consent process for DCD with respect to disclosure of necessary elements for voluntary informed decision making and respect for the donor's autonomy. We compare two alternative proposals for increasing organ donation consent in society: presumed consent and mandated choice. We conclude that proceeding with the recovery of transplantable organs from decedents requires a paradigm change in the ethics of organ donation. The paradigm change to ensure the legitimacy of DCD practice must include: (1) societal agreement on abandonment of the dead donor rule, (2) legislative revisions reflecting abandonment of the dead donor rule, and (3) requirement of mandated choice to facilitate individual participation in organ donation and to ensure that decisions to participate are made in compliance with the societal values of respect for autonomy and self-determination.

## Background

Medical and pharmacologic advancements have made it possible to transplant organs successfully and thereby to save the lives of many persons who otherwise would die from irreversible end-stage organ disease. The greatly enhanced technical ability to transplant organs has also led to an ever-increasing need for transplantable organs [1]. The explosive growth in the demand for and the marginal increase in the supply of transplantable organs have together been characterized as an 'evolving national health care crisis' [2]. In fact, organ donation rates nationally have changed little in the past 15 years [3], whereas the need for donated organs has grown 5 times faster than the number of available cadaveric organs [4]. It is therefore no surprise that the transplantation community and society as a whole now consider balancing the demand for and the supply of transplantable organs as one of their biggest challenges.

The continually increasing need for organs led to the reintroduction of the principle of donation after cardiac or circulatory death (DCD) in the early 1990s with the Pittsburgh protocol to complement already available organ procurement from brain-dead persons [5,6]. A new federal mandate requires hospitals as of January 2007 to design policies and procedures for organ procurement in DCD to increase the rate of organ donation and recovery from decedents to 75% or greater [7-9].

However, DCD is controversial because of medical, ethical, and legal uncertainties about the premise that donors are indeed dead before their organs are procured [10-13]. In this article, we contend that the recovery of viable organs useful for transplantation in DCD is not compatible with the dead donor rule and we explain the ethical and legal ramifications of DCD. We also examine the current process of consent for organ donation and whether it includes the necessary elements for voluntary informed consent (i.e., the full disclosure of information relevant to decision making and respect for the person's autonomy). We will contrast the ethical aspects of two alternative proposals for increasing donation consent in society: presumed consent and mandated choice. Finally, we will conclude by positing that in order for the current principle of DCD to proceed with recovery of transplantable organs from decedents, a paradigm change in the ethics of organ donation is necessary. The paradigm change to ensure the legitimacy of DCD practice must include (1) societal agreement on abandonment of the dead donor rule, (2) legislative revisions reflecting abandonment of the dead donor rule, and (3) the requirement of mandated choice to facilitate individual participation in organ donation and to ensure that DCD is in compliance with the societal values of respect for autonomy and self-determination.

## DCD and the dead donor rule

The criteria for determining death play a prominent role in the acceptability of DCD. The recovery of viable organs for successful transplantation must be achieved with the donor already dead at the time of procurement in order to comply with the dead donor rule. Whereas some have considered a person dead after 2 minutes of apnea, unresponsiveness, and absent arterial pulse [5], the Institute of Medicine recommended waiting for 5 minutes of absent consciousness, respiration, and mechanical pump function of the heart (zero pulse pressure through arterial catheter monitoring), irrespective of the presence of electric activity of the heart (evident on electrocardiographic monitoring) [14]. In 2001, the American College as well as the Society of Critical Care Medicine concluded in a position statement that a waiting period of either 2 minutes or 5 minutes was physiologically and ethically equivalent and therefore either was an acceptable timeline for beginning the process of organ retrieval [15]. Waiting for longer than 5 minutes can cause warm ischemia and detrimentally affect the quality of procured organs and impair their suitability for transplantation. However, critics have argued more than a decade ago that the waiting time to determine death by respiratory and circulatory criteria is based on insufficient scientific evidence [10]. The spontaneous return of circulation and respiration (i.e., the Lazarus phenomenon or autoresuscitation) has been reported to occur in humans as long as 10 minutes after cessation of circulation and respiration. Autoresuscitation appears to validate previous concerns that viable organs may be procured from persons who are in the process of dying yet are not truly dead [16-18].

According to the Uniform Determination of Death Act (UDDA) of 1981, a person is determined dead after having sustained either irreversible cessation of circulatory and respiratory functions or irreversible cessation of all brain function, including that of the brain stem, and the determination of death must be made in accordance with accepted medical standards [19]. The President's Commission for the Study of Ethical Problems in Medicine and Biomedical and Behavioral Research defined the statute for the determination of death so that ""Death is a Single Phenomenon" [20]. The statute is intended to address the question "how, given medical advances in cardiopulmonary support, can the evidence that death has occurred be obtained and recognized". The President's Commission defined the cessation of circulation to be irreversible for death determination " [i]f deprived of blood flow for at least 10–15 minutes, the brain, including the brainstem, will completely cease functioning". A 4–6 minute loss of blood flow – caused by, for example, cardiac arrest – typically damages the cerebral cortex permanently, while the relatively more resistant brainstem may continue to function."

The challenge in determining death for organ procurement is twofold: (1) the use of an arbitrary set of criteria and time frames to define irreversible cessation of circulatory and respiratory functions without evidence of the uniformity for death determination and (2) the variability of the criteria used by different institutions for organ procurement protocols [14,21].

The notion of irreversibility of cessation of circulatory and respiratory functions has been a contentious medical and ethical issue. Tomlinson proposed a definition of irreversibility as "a requirement that arises only at the level of the *criteria for the determination *of death, rather than at the level of the concept of death, just as 'beyond reasonable doubt' is not a part of the *concept *of 'guilty', but instead is a requirement for the legitimate determination of guilt within a judicial system." [22]. The requirement for irreversibility therefore depends on the context in which, and the purposes for which, the concept of death is being used [22]. The notion of irreversibility is commonly understood as meaning either that the heart cannot be restarted spontaneously (a weaker construal) or that the heart cannot be restarted despite standard cardiopulmonary resuscitation (a stronger construal). The stronger construal of irreversibility as meaning "can never be reversed" implies in its extreme that *at no time *can organ procurement ever be permissible because future possibilities of resuscitation can never be fully ruled out. In practical terms, the weaker definition of "not reversible now" implies that a person is considered irreversibly dead based on that person's moral choice to forego resuscitative interventions; thus, as long as the probability of autoresuscitation is negligible, the dead donor rule is not violated. On the basis of that argument, the notion of irreversibility depends on the person's choice to forego resuscitative interventions after spontaneous cessation of circulatory and respiratory functions. However, the argument that irreversibility can be understood as a moral choice is flawed. First, the issue is not whether there are good reasons not to resuscitate a person but whether the person is truly dead [18]. Second, resuscitative interventions are performed during the procurement process to keep organs viable for transplantation after the cessation of vital functions. The use of artificial cardiopulmonary bypass machines, external mechanical cardiac compression devices, and reinflation of the lungs to preserve organs for procurement also results in the resuscitation of the heart and the brain after the formal declaration of death. Resuscitation of the brain with a return of consciousness is particularly problematic because the Institute of Medicine announced in its 2006 report that expansion of the organ donor pool by procuring organs from living persons with normal brain function who sustain sudden cardiac death is morally acceptable [23].

Longer than 10 minutes of absent circulation is required for irreversible cessation of the entire human brain, including brain stem function. The administration of medications to suppress heart and brain functions is therefore required when the procurement process begins within 5 minutes of cessation of circulation [12,24].

The use of resuscitative methods and medications to suppress heart and brain functions during organ procurement raises a host of additional ethical and legal questions. Organ donors consent to the withholding of all resuscitative interventions after cessation of circulatory and respiratory functions through a do-not-resuscitate (DNR) directive. Under such conditions, the use of resuscitative methods for organ procurement violates not only the dead donor rule but also the person's health directives. The strong probability of a return of heart and brain functions during procurement also means that the act of organ removal is the immediate and proximate cause of death for that person.

The need for criteria to sharpen "the indeterminate boundary between life and death" for death determination has been widely recognized [25]. The dependence on both circulatory and respiratory criteria only for the determination of death in DCD is problematic and conceptually inconsistent because of (1) there is a likelihood of spontaneous reversibility of circulatory and respiratory functions when organ procurement begins, and (2) the possibility for the brain to recover function long after circulatory arrest, particularly when artificial circulation is used for organ procurement. Therefore, the practice of DCD conflates a prognosis of death with a diagnosis of death [12,26]. The application of criteria for irreversible cessation of neurologic, circulatory and respiratory functions requires a waiting time well in excess of 10 minutes to sharpen the determination of death for organ procurement [27-32]. However, that waiting time can also make it more difficult to recover viable organs for transplantation. The simultaneous determination of total cessation of the activity of the entire brain, including the brain stem, is required for the determination of death when respiration and circulation are artificially supported during organ procurement. Capron and Kass emphasized in the President's Commission when defining death "A person will be considered dead if in the announced opinion of a physician, based on ordinary standards of medical practice, he has experienced an irreversible cessation of respiratory and circulatory functions, or in the event that artificial means of support preclude a determination that these functions have ceased, he has experienced an irreversible cessation of total brain functions"[20].

## The dead donor rule and the law

DCD has been recommended on the basis of the utilitarian rationale of maximizing the number of organ transplants in order to save more lives. This utilitarian approach has also provided implicit justification for manipulation of some aspects of the death process [33]. Intervention has been justified not only in the dying process but also in defining the word *dead*. The uncertainty of the uniformity of determination of death in DCD has legal implications [34]. The act of procurement or the removal of organs from persons who may still be in the process of dying but who are labeled as being dead, becomes the direct and proximate cause of death or of "killing" rather than the natural illness itself [35]. Medically redefining death arbitrarily to permit DCD for organ procurement has been a necessary prerequisite for the circumvention of homicide law. Declaration of death or calling someone dead takes the burden off procurement personnel and provides the appearance that it is acceptable to remove organs under such conditions without being found guilty of murder [36]. The purposeful manipulation of the criteria for the determination of death serves the desired goal of increasing the opportunities for procurement of transplantable organs, but it also represents a knowing gerrymandering of the existing legal definition [34]. The President's Commission indicated in the 1981 report on defining death that the UDDA is intended to aid in the process of recognition and providing a legal standard to distinguish the dead from the dying and, ought not to reinforce the misimpression that there are different "kinds" of death, defined for different purposes, and hence that some people are more "dead" than others[20]. An argument can be made that a person's consent or permission for organ donation can legitimize this intervention, as with any other medical procedure with potential risk of death. However, that argument transgresses the legal limits of autonomy, because no person can consent to his or her own killing. The ban on assisted suicide, regardless of a person's wishes, reaffirms that society has a consolidated interest in preserving life. In the United States (U.S.), physician-assisted suicide is legalized only in the State of Oregon.

## Problems with consent for organ donation

Organ procurement organizations (OPOs) are the designated requesters for organ donation [37,38]. Hospitals are required to notify OPOs of all imminent deaths before withdrawal of ventilator support to allow OPO representatives to initiate independent discussion of consent for organ donation with surrogates [7,39]. The OPOs are private organizations under government contract with Medicare and Medicaid Services to coordinate deceased organ procurement [7]. Each OPO has significant financial incentives for maximizing organs recovery through consent for donation from hospitals located within the donation service area. The Organ Donation Breakthrough Collaborative has set three top-level goals for each OPO to achieve: 1) a 75% or higher organ donation (or conversion) rate from regional hospitals, 2) 3.75 (or greater) organs transplanted per organ donor and 3) DCD to account for 10% (or greater) of donation service area's deceased donors, without a decrease in brain dead donors [37]. The successful compliance with the set goals are required for each OPO to maintain active certification and renewal of contract with Medicare for payment for services provided in a donation service area [7]. Additional financial incentives for the OPO to aggressively purse organ donation in Medicare approved hospitals include reimbursement for actual donors, financial returns on local transplant activity solely supported by local donor activity and Medicare incentives for local organ donation activity [40].

Obtaining consent is considered one of the guiding principles that provide moral validation of organ transplant programs. Consent for organ donation can be registered and documented in several ways. The donor registry is an online electronic database for accessing donor consent information and it is readily available to OPO personnel. In contrast, donor consent documented on driver's licenses, donor cards, or advanced directives may not be available to clinicians when donation or procurement decisions must be made [41]. Consent for organ donation is obtained in two different situations. The first situation is to acquire consent from healthy persons for future organ donation. It is generally achieved by inviting members of the public to complete donor cards (e.g., as part of a driver's license application) providing general consent for organ donation or to consent to organ donation by signing up on a state registry when they visit an OPO Web site [42]. The second situation occurs when consent is obtained from a surrogate decision maker for a brain-dead person or a person for whom death is imminent and who has not expressed intent for organ donation through a driver's license, a donor card or donor registry.

Studies show that half of the families who are asked to consider donation after a relative's death refuse consent [43]. It should therefore come as no surprise that in addition to educating the public, the Institute of Medicine Committee on Increasing Rates of Organ Donation has identified among its primary objectives an increase in the number of opportunities for people to record the decision to donate and the enhancement of donor registries to ensure full access to and sharing of donor registration data [23].

Requiring consent is consistent with one of the cornerstones of medicine and bioethics: respect for individual autonomy. Among other things, the process of obtaining consent must include the provision of an appropriate quantity and quality of information so that the person can make an informed decision. Currently, the consent for DCD is requested with disclosure of similar information as with brain-death donation. Given the medical and ethical uncertainties surrounding DCD, its consent process should be expected to be different from that used in brain-death donation. The differences between the two types of organ donation with regard to timing and the nature of the procurement procedure, nonbeneficial interventions, and trade-offs in end-of-life care are not often clarified to potential donors or surrogate decision makers at the time of consent [44]. DCD also exposes donors to the risk of failing to die within the allotted time frame for successful organ procurement after the performance of predonation procedures [45].

Considering that actual donation or procurement processes differ according to the death criteria, one might expect the consent process to include details about the various death scenarios. In 2006, Woien et al examined the quality and quantity of information about consent that is disclosed to the public and to potential organ donors on OPO Web sites [44]. The information content about relevant aspects of medical interventions, procedures, protocols and changes to the quality of end-of-life care was found to be deficient because it was focused primarily on the encouragement and reinforcement of consent to donation [44]. This lack of disclosure on OPO Web sites and in online consent documentation raises doubts about whether organ donors actually receive and understand the pertinent information necessary to making an informed decision about whether to participate in deceased organ donation. The lack of detailed and accurate disclosure violates the tenet of informed consent and abuses the public's trust in the deceased organ donation system.

The medical community is expected to be transparent and to fully inform the public about the different donation practices and their implications. Yet, disclosing more detailed information about organ donation to the general public may very well result in a decrease in donor registrations [46]. Suggestions that the organ supply shortage is a health care crisis may also have a detrimental effect by exacerbating public fears and by fueling excessive worry or speculation that procurement decisions may ultimately go beyond socially accepted thresholds. The Institute of Medicine has proposed changes in the consent format as a way to increase the organ donation rate in the community while also reducing the risk of increased public fear [23]. The explicit or express consent of competent adults or surrogate decision makers is the current standard for organ donation consent. Other consent options include the presumed consent, conscription (sometimes referred to as routine removal) or mandated choice.

## Presumed consent

Presumed consent means either implied consent inferred from other actions or tacit consent that constitutes consent in the absence of explicit dissent [47,48]. Presumed consent within the context of organ donation implies a default position of donation for those persons who do not take action to dissent (opting out). The switch from *explicit *consent to *implicit *presumed consent has been advocated as an efficient method to increase the supply of transplantable organs.

The ethical justification commonly given for a switch to presumed consent is twofold. First, polls show that about 69% of Americans are "very likely" or "somewhat likely" to grant permission to have their organs harvested after death, [43] which suggests broad public support. However, there has always been a gap between people's perceived attitudes in polls on organ donation and what they do in practice. Perhaps this is not simply a refection of knowledge but of personal experiences and beliefs [49]. Also in a subsequent national survey of organ and tissue donation attitudes and behaviors (conducted by the Gallup Organization and prepared for the Division of Transplantation Health resources and Services Administration), most people either "opposed" (26.7%) or "strongly opposed" (30.1%) presumed consent [50]. In the same survey, about 3 in 10 reported that they would opt-out of a presumed consent approach. Second, as some have argued, deceased organ donation should be considered a duty rather than an act of charity [51]. Hester postulated that "deciding not to release our organs for transplantation would constitute a serious moral wrong" in light of the desperate need for transplantable organs [52].

Presumed consent certainly poses a challenge to the principle of protecting a person's right to fully informed agreement (consent), and its moral justification therefore falls short. First, the issue of a moral obligation to donate organs at death is still subject to debate; a public discourse on this topic has not yet taken place. Second, access to health care including organ transplantation services is not universal. Data released in August 2006 by the U.S. Census Bureau showed that more than 46.8 million people are uninsured and 24.4% of those earned less than $25,000, an unknown number of people had limited health care coverage, and 12.6% of the U.S. population lived below the poverty margin [53]. As the erosion of employer-based health insurance continues, the numbers of underinsured and uninsured persons are likely to increase. In addition, 82% of kidney recipients are white which leads one to speculate that there may be racial discrepancy in organ allocation [54]. Third, the duties of relevant stakeholders in health care remain poorly defined. The question of who is responsible for what in health care has yet to be answered, which is even more troubling in light of the fact that health care in the 21st century is more commonly understood solely in terms of a commodity operating in a self-regulating free-market environment. How complex social interactions are to be arranged is a subject of rational discourse for which every participant should assume responsibility and be held accountable [55].

Widespread public education and clear, easy and transparent ways for persons to register dissent are requirements for an ethically acceptable presumed consent policy [23]. Considering that the current process of donation consent is deficient in its provision of basic information about organ donation and that there is an absence of established social practices that would warrant the presumption of consent for organ donation, the justification is lacking for a switch from express to presumed consent in the United States.

Conscription, also referred to as mandatory donation, is the routine postmortem removal of organs for transplantation. As such, it presupposes society's right of access to the organs of any deceased person. Such a right would rest either on the claim that society "owns" the body of the deceased or on the premise of an enforceable moral duty all of us as humans have to allow postmortem organ retrieval. In the U.S., the government does not claim complete authority over the disposition of the bodies of the deceased [23]. Some states in the U.S. have even interpreted the right of a person or family to decide whether to donate organs as an interest sufficient to endow some rights to the corpse that cannot be disregarded without due process. Such laws have assigned a property interest in the body to the next of kin [56]. Conscription would depart from this legal principle as well as from the norm of expressed consent.

Although the routine removal of organs after death is inconsistent with current U.S. federal and state laws, some proponents postulate the appropriateness of conscription on practical and ethical grounds. Practical arguments include the fact that people with organ failure are dying daily because of the short supply of transplantable organs and that many usable organs are never made available, most commonly because of family refusal. Conscription would override family refusal for donation and produce an efficient rate of deceased organ recovery almost close to 100% [57]. Conscription would eliminate the need for costly public education programs, training of requesters, and maintenance of donor registries; it might also alleviate concern about abuse or possible commodification of the human body. The duty-based justification for conscription fails, however, because organ transplantation practices are inconsistent with the requirement of universality. Not everyone is included in a fair system that is mutually beneficial. Conscription would maximize organ recovery but would do so to the detriment of respect for personal autonomy and accepted societal norms. It would also violate the religious values of some persons for the body to not be buried whole [58].

## Mandated choice

The second consent option is that of mandated choice. Mandated choice would require all adult persons in the community to consider organ donation and to document their decision. All competent adults would be required to decide in advance to agree to organ donation or to refuse organ donation, and their wishes would be considered legally binding (unless they had a documented change of mind before actually dying). Mandated choice would preserve altruism and the voluntary nature of donation, and as such proponents consider it to be consistent with the principle of respect for autonomy [59]. Opponents of mandated choice postulate that it is unacceptable in a libertarian society to force people to make choices [60] and that mandated choice is coercive and an intrusion on privacy [61]. Concerns have also been raised that mandated choice would disallow consideration of the views of the family [62].

With the current view of the shortage of transplantable organs identified by many people as a health care crisis, one might argue that neither a mandate to make an autonomous prospective decision about organ donation nor the expectation of a family's compliance with the wishes of the deceased is unreasonable. A similar justification can be made about the intrusion of privacy associated with mandated choice [63]. However, mandated choice would require full disclosure of relevant unbiased information about all aspects of organ procurement that, in turn, constitutes informed consent. The importance of public education in mandated choice is illustrated by the failure of a state initiative in Texas. In 1991, Texas enacted a law requiring citizens to make a "yes" or "no" choice about organ donation when they renewed their driver's license. The law had to be repealed in 1997 because the implementation of the mandatory choice resulted in a refusal rate of 80% [64]. This high rate of refusal was attributed to the lack of public education about organ donation [65]. It is therefore of great concern that OPOs today have focused their efforts on convincing members of the public to become organ donors rather than on providing adequate unbiased information and education about organ donation. A 2006 report from the Institute of Medicine suggested that optimal public education would be cost prohibitive and labor intensive [23].

## Paradigm transformation of organ donation ethics

There is growing doubt among scholars and medical practitioners that DCD can comply with the principles on which it was introduced into society as an ethically acceptable practice. We have highlighted several concerns indicating that the current DCD practice not only violates the dead donor rule but also puts the moral legitimacy of consent for donation in question. Unless the current DCD practice is reevaluated, the erosion of public trust and damage to the integrity of the medical profession are likely to develop over time. To avoid these negative consequences, we are faced with implementing any or all of three strategic options. The first strategy would be to discontinue DCD and instead focus on reducing the demand for transplantable organs by promoting healthy lifestyles (i.e., primary and secondary prevention programs for chronic diseases such as diabetes and hypertension) [66,67]. This strategy might decrease the future incidence of end-stage organ disease and the resulting need for transplantation; however, it would not resolve the current imbalance between the supply of and the demand for organs. The second strategy would be to revise the uniform definition of death to allow the definition of "dead" to be applied to dying persons so that the recovery of transplantable organs from DCD can be continued in an ethical and legal manner [36]. Bernat, for instance, has argued for a change in the standard determination of death that would substitute "permanence" for "irreversibility" and thereby permit the classification of dying persons as truly dead [68]. Bernat's proposal to change the death determination implicitly acknowledges that the current DCD practice is inconsistent with the dead donor rule. Bernat justifies violation of the dead donor rule and there is no need to distinguish between the "dying" and the "dead" for the purpose of organ procurement for transplantation. The justification put forward by Bernat conflicts with the President's Commission views on when and how the death statute is applied "to distinguish the dead from the dying" and to prevent "the mistaken impression that a special "definition" of death needs to be applied to organ transplantation, which is not the case" and that it "ought not to reinforce the misimpression that there are different "kinds" of death, defined for different purposes, and hence that some people are [more dead] than others" [20].

The word "permanence" conveys the absolute accuracy of the "prognosis" rather than a determination or diagnosis of death. However, opponents of the criterion of absolute certainty of prognosis of death may consider as homicide its application to persons for whom the consent to withdraw artificial life support is made [69]. Revising the UDDA in this manner would have far-reaching ethical implications not only for society but also for criminal and homicide laws. Criminal prosecution, inheritance, taxation, treatment of cadaver, and mourning are all affected by the way society draws the dividing line between life and death [20]. More importantly, it can violate the principle of nonmaleficence by allowing the introduction of errors in prognostication that may have a detrimental effect on end-of-life care and palliation. The third strategy would be to abandon the dead donor rule for organ procurement so that procuring organs becomes permissible during the terminally ill person's dying phase after voluntary informed consent has been obtained [26]. The abandonment of the dead donor rule would constitute a paradigm switch in the ethics of deceased organ procurement for transplantation from donor beneficence to autonomy and nonmaleficence. Donors would be solely responsible for their decisions, and the medical community would have to comply with the do-no-harm principle at the end of life. As is the case with revising the determination of death, this paradigm switch would require changes in criminal and homicide laws to legitimize DCD legally, ethically, and medically. In addition, changing the paradigm would require public discourse about permitting autonomy-based end-of-life decisions. The preservation of a person's autonomy and the voluntary nature of the decision are fundamental for such a profound paradigm shift and, as such, they require comprehensive public education and disclosure of all relevant information. The mandated personal choice in conjunction with the paradigm shift would protect an individual's right to agree or refuse and thereby would eliminate coercion in the organ donation consent process with minimal infringement on privacy. Within this context, mandated choice restores the public trust and eliminates the individual's fear of manipulation of the dying and death process for the intent of organ procurement. Mandated choice is compatible with the principle of respect for individual autonomy and decision making, and it does not require additional consent from a person's family to procure organs after death.

## Conclusion

The long-term solution for overcoming the shortage of transplantable organs is to focus on, and to broadly implement, universally accessible preventive health-care programs. For the short term, increasing the number of potential donors while also maintaining the public trust and the integrity of medicine requires public education, a consent process characterized by full disclosure of relevant information about organ donation and procurement procedures critical to the decision making about organ donation, and a switch of the ethics paradigm from beneficence to nonmaleficence and respect for individual autonomy to allow for DCD to comply with legal and ethical standards. The implementation of mandated choice for obtaining consent would appear reasonable and morally justifiable to assist with the objective of increasing the number of people who consent to organ donation after death. Ultimately, the outcome of public debate must be the decisive factor in determining the conditions under which DCD should be considered legitimate.

## Abbreviations

DCD, donation after cardiac death, donation after circulatory death

UDDA, Uniform Determination of Death Act

U.S., United States

OPO, organ procurement organization

## Competing interests

The authors have no affiliations or financial involvement with any organization or entity with a direct financial interest in the subject matter or materials discussed in this manuscript. The authors have no financial or non-financial competing interests to disclose.

## Authors' contributions

The authors (J.L.V., M.Y.R., and J.M.) attest that they have each made substantial contributions in drafting the manuscript or revising it critically for important intellectual content; that they have given final approval of the version to be published; and that they have participated sufficiently in the work to take public responsibility for appropriate portions of the content. J.L.V., M.Y.R., and J.M. have all read and approved the final manuscript.
